# New indices from microneurography to investigate the arterial baroreflex

**DOI:** 10.14814/phy2.13220

**Published:** 2017-06-29

**Authors:** Alexandre Laurin, Matthew G. Lloyd, Tesshin Hachiya, Mitsuru Saito, Victoria E. Claydon, Andrew Blaber

**Affiliations:** ^1^ Department of Biomedical Physiology & KinesiologySimon Fraser UniversityBurnabyBritish ColumbiaCanada; ^2^ InriaUniversité Paris‐SaclayPalaiseauÎle‐de‐FranceFrance; ^3^ LMSÉcole PolytechniqueCNRSUniversité Paris‐SaclayPalaiseauÎle‐de‐FranceFrance; ^4^ Department of Aerospace PsychologyNagoya UniversityJapan

**Keywords:** Baroreflex, microneurography, signal analysis

## Abstract

Baroreflex‐mediated changes in heart rate and vascular resistance in response to variations in blood pressure are critical to maintain homeostasis. We aimed to develop time domain analysis methods to complement existing cross‐spectral techniques in the investigation of the vascular resistance baroreflex response to orthostatic stress. A secondary goal was to apply these methods to distinguish between levels of orthostatic tolerance using baseline data. Eleven healthy, normotensive males participated in a graded lower body negative pressure protocol. Within individual neurogenic baroreflex cycles, the amount of muscle sympathetic nerve activity (MSNA), the diastolic pressure stimulus and response amplitudes, diastolic pressure to MSNA burst stimulus and response times, as well as the stimulus and response slopes between diastolic pressure and MSNA were computed. Coherence, gain, and frequency of highest coherence between systolic/diastolic arterial pressure (SAP/DAP) and RR‐interval time series were also computed. The number of MSNA bursts per low‐frequency cycle increased from 2.55 ± 0.68 at baseline to 5.44 ± 1.56 at −40 mmHg of LBNP. Stimulus time decreased (3.21 ± 1.48–1.46 ± 0.43 sec), as did response time (3.47 ± 0.86–2.37 ± 0.27 sec). At baseline, DAP‐RR coherence, DAP‐RR gain, and the time delay between decreases in DAP and MSNA bursts were higher in participants who experienced symptoms of presyncope. Results clarified the role of different branches of the baroreflex loop, and suggested functional adaptation of neuronal pathways to orthostatic stress.

## Introduction

Baroreflex‐mediated changes in heart rate and vascular resistance in response to variations in blood pressure are critical to maintain homeostasis. Inadequate responses may contribute to orthostatic hypotension and vary widely in severity, causes, and symptoms (Moya et al. 2009). In fighter pilots and astronauts, orthostatic hypotension due to severe changes in gravitational forces can cause an interruption in cognitive function and syncope, which can be devastating during mission‐critical procedures (Scott et al. 2007; Watenpaugh and Hargens 2011). Elderly individuals have a high prevalence of orthostatic intolerance and orthostatic hypotension, and this has been linked to higher risks of myocardial infarction (Luukinen et al. 2004), stroke (Eigenbrodt et al. 2000), and may also cause syncope, a significant contributor to falls and injury (Lipsitz et al. 1986).

Baroreceptors located in the carotid sinus, coronary arteries, and the aortic arch respond to changes in vessel diameter. Decreased blood pressure reduces the distention of vessel walls, which in turn decreases baroreceptor afferent activity. This disinhibits the vasoconstrictor center of the medulla, increasing sympathetic activity, and inhibiting the vagal parasympathetic center. The net effects in the heart are decreased heart rate and contractility. The effect on the peripheral blood vessels is general constriction.

It has been shown in young adults that an impairment in the ability to increase vascular responses to baroreflex stimulation when upright is associated with poor orthostatic tolerance (Cooper and Hainsworth 2002). This has led to the hypothesis that maintenance of blood pressure during orthostasis depends primarily on a vascular response, and second on a cardiac response (Brown and Hainsworth 2000; Claydon and Hainsworth 2004). However, despite extensive evidence of the relative importance of vascular responses (Waters et al. 2002; Wieling et al. 2014), some debate remains regarding the relative contribution of the cardiac and vascular responses to orthostasis (Convertino 2014). This may be perpetuated in part by the relative ease of examination of cardiac compared to vascular responses.

Previous work has shown that the frequency of maximal coherence between RR interval and systolic arterial pressure time series in the low‐frequency range was correlated to time to presyncope in individuals with histories suggestive of unexplained syncope (Gulli et al. 2001, 2003). Due to its minimal invasiveness, and because it can be obtained at rest, this index holds great potential to help in the clinical diagnosis of orthostatic intolerance. The physiological interpretation of this observation is difficult to obtain, however, in part due to the absence of concurrent direct vascular analyses in the cited studies.

**Figure 1:**
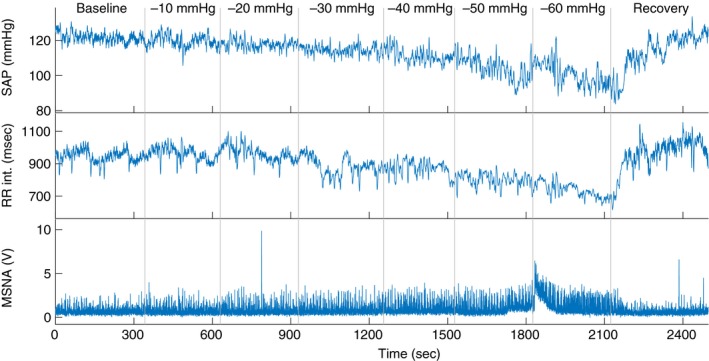
An example of typical systolic arterial pressure (SAP), RR intervals, and muscle sympathetic nervous activity (MSNA) responses during graded lower body negative pressure (LBNP). The participant shown here did not experience presyncope.

Lower body negative pressure (LBNP) is a technique where a participant's lower body is placed in an airtight box at the level of the iliac crest, and air is removed via a vacuum pump to reduce the internal box pressure. It is often used to investigate the reaction of the cardiovascular system to stress similar to standing or hypovolemia, but with minimal muscle pump effect and no vestibular stimuli (Stevens and Lamb 1965; Hachiya et al. 2010). The technique pools blood into the lower body, reducing blood pressure and subsequent baroreflex responses can be determined (Fig. 1).

While the parasympathetically mediated effects on heart rate are easily measured via surface electrocardiogram, quantification of sympathetic outflow is more difficult. Microneurography has been applied in laboratories since the late 1960s to record muscle sympathetic nervous activity (MSNA) and quantify sympathetic outflow to the vasculature (Hagbarth and Vallbo 1968). The technique has recently been used to study the arterial response to changes in blood pressure and its modulation during presyncope in orthostatically tolerant individuals (Iwase et al. 2002; Hachiya et al. 2012). Although these studies have contributed significantly to our understanding of the relationship between blood pressure and MSNA before and during presyncope, the challenge remains to understand the relative contributions to orthostatic tolerance of both cardiac and arterial branches of the baroreflex.

Blood pressure, heart rate, and MSNA all contain a low‐frequency oscillation at ~0.1 Hz, and they are in large part due to inherent time delays in the processes involved in the centrally mediated baroreflex (Mayer 1876; Julien 2006). Communication latency between receptor and effector organs in the baroreflex‐negative feedback loop causes constant over/undershoot and readjustment at the resonant frequency of 0.1 Hz. Studying the relationships between the components of low‐frequency (LF) oscillations is key to understanding the baroreflex. Low‐frequency DAP and MSNA fluctuations, as well as the relationship between these two variables, are usually quantified by frequency analysis. Individual DAP and MSNA power quantifies the amount of activity during a given period of time, and coherence quantifies the degree to which their oscillations are related. In normotensive patients at rest, DAP and MSNA power in the LF range is relatively low, as is their coherence. Upon application of LBNP, coherence increases, as do the respective powers in the LF range (Furlan et al. 2000; Cooke et al. 2009). This likely reflects baroreflex engagement during orthostatic stress, even when that stress is minimal, such as during a sit‐to‐stand test or a minute of −10 mmHg of LBNP. Although spectral and cross‐spectral analyses have been used for years to study the relationships between RR intervals, blood pressure, and MSNA, gaps exist in their ability to offer a complete picture of the variables at play. Specifically, it remains difficult to quantify the cause and effect of individual sequences of MSNA bursts, as well as measure the various time delays involved.

The primary aim of this study was to develop analysis methods to investigate the vascular resistance baroreflex response to orthostatic stress. The secondary aim was to investigate the application of the new analysis methods to predict levels of orthostatic tolerance from baseline data.

## Methods

### Ethical approval

Experiments were conducted in accordance with the Declaration of Helsinki and with approval from the Simon Fraser University Office of Research Ethics. All participants provided written informed consent.

### Protocol

Eleven healthy, normotensive male participants (average age 21 ± 1 years) agreed to partake in a graded LBNP protocol that had the following progression of pressures: 0, −10, −20, −30, −40, −50, and −60 mmHg. Each level was sustained for 5 min. If a participant exhibited a sudden decrease in heart rate or blood pressure, or if they expressed a desire to stop, the negative pressure was immediately terminated.

Participants that did not experience presyncope were classified as finishers (*n* = 6). Those that displayed presyncope before completing the 5‐min −60 mmHg level were classified as nonfinishers. For nonfinishers, symptoms of presyncope occurred either during the −50 mmHg level (*n* = 4) or at the onset of the −60 mmHg level (*n* = 1).

Continuous measurements of ECG, MSNA, and blood pressure were recorded simultaneously on a personal computer with analog‐to‐digital conversion (Acqknowledge, Biopac Systems, Goleta, CA) for subsequent analyses.

### Hemodynamic monitoring

A continuous analog lead II electrocardiogram signal (model information) was recorded and used to obtain R waves using the Pan–Tompkins algorithm (Pan and Tompkins 1985). RR intervals were then computed.

Continuous waveform blood pressure was determined using finger photoplethysmography (Finapres, Ohmeda, Englewood, CO), calibrated to automatic electrosphygmomanometry performed at baseline (Nippon Colin BP 203, Tokyo, Japan). Systolic and diastolic arterial pressures (SAP and DAP) were obtained as the maximum and minimum arterial pressures in each RR interval.

Time series were generated for the RR interval, DAP, and SAP data (time difference between the R peak and diastolic timing was subtracted from both systolic and diastolic timings). The time series were resampled at 5 Hz using a shape‐preserving piecewise cubic interpolation method and forward–backward band‐pass filtered to a band of 0.04–0.15 Hz to isolate LF oscillations. This filter design, particularly in its application in one time direction and then the other, was chosen to minimize its effect on phase.

Cross‐spectral analyses of the RR interval and SAP time series were also performed. These time series were fitted with a bivariate autoregressive model (Bartoli et al. 1985). Since our aim was to identify oscillations of ~0.1 Hz in a signal sampled at 5 Hz, a model order of 100 was chosen. Coherence, frequency, and transfer function gain (cardiac baroreflex sensitivity) between SAP and RR were computed at the point of maximal coherence in the LF range, where phase was negative (indicating that changes in blood pressure preceded the changes in RR interval) (Taylor et al. 1998; Cevese et al. 2001). Gain and frequency values were only accepted when coherence was above 0.5.

### Microneurography

MSNA signals were obtained by microneurography using techniques described previously (Hachiya et al. 2008). Bursts were identified as points having a prominence of at least three times the interquartile range (3×IQR) of the MSNA signal, and occurring between 0.8 and 1.5 sec after an R wave.

To isolate single neurogenic constriction–dilation cycles in the LF band, peaks were identified on the filtered and resampled DAP signal as any positive relative maximum with at least 6.67 sec (or 1/0.15 Hz) between them (the smallest physiologically possible distance between two successive LF peak events). A point was identified as a local maximum in the simplest possible sense, that is, if it was higher than both the directly previous and directly successive points. These peaks were then used to obtain the closest relative maximum on the unfiltered DAP signal, which in turn were used as estimates for the time of maximum constriction. The identified maxima were then used as cutoffs between cycles and the segments between them were then deemed single LF cycles in both DAP and MSNA. A similar technique of extrema identification in filtered blood pressure time series has been used to quantify pain during surgery (Logier et al. 2006), and to compute phase difference between SAP and RR intervals (Mitrou et al. 2017).

It is known that acute decreases in DAP cause increased MSNA (Sundlöf and Wallin 1978). In every LF cycle, the steepest decrease in DAP was identified to estimate the time of DAP‐mediated sympathetic activation. For each cycle, MSNA bursts occurring between the steepest decrease and the end of the cycle were identified.

**Figure 2:**
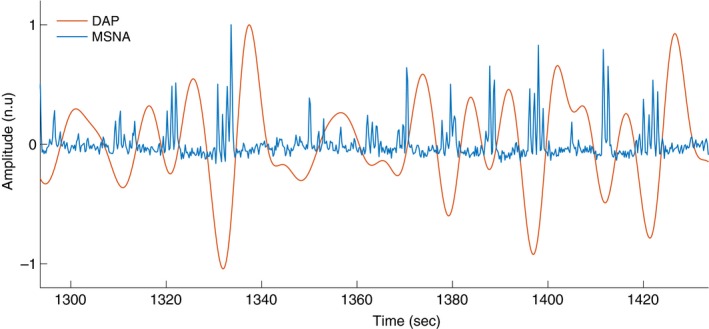
An example of concurrent MSNA and DAP in normalized units. DAP was filtered to a band‐pass of (0.04, 0.15) Hz. Some individual LF baroreflex cycles are clearly identifiable as beginning with a decrease in DAP followed by one or more bursts of MSNA, followed in turn by an increase in DAP. During other periods, the relationship between the two variables seems less strong. The strength index *S* established criteria to automatically select the “coupled” cycles.

Many variables affect MSNA and DAP. To locate the LF cycles where the DAP–MSNA relationship was strongest, an estimator of strength (S) for each cycle was obtained. *S* was computed as the multiplication of MSNA area, number of MSNA bursts, and DAP amplitude. The strength index *S* was inspired by coherence, which measures the degree to which oscillations over a frequency band in one signal correspond to those in another signal in single cycles. We then designed an index that favored clear, high‐amplitude LF DAP cycles that contained high MSNA in their center (Fig. 2). Because the MSNA area can be influenced by factors unrelated to neural traffic, such as electrode placement, this estimator was not used for comparisons between participants. The 10 cycles with the highest *S* at each LBNP level were kept for further analysis. While 10 cycles were chosen for analysis in this study, use of 5–15 cycles yielded equivalent results. Incorporation of more than 15 cycles became problematic at baseline or low levels of LBNP, where aberrant single LF DAP cycles could be incorporated that had negligible amplitudes and/or irregular shapes.

**Figure 3:**
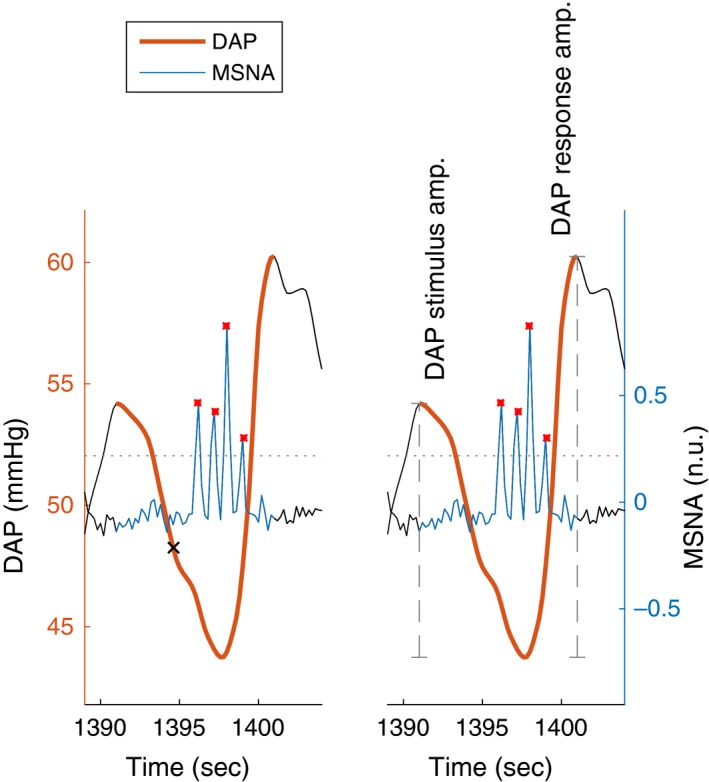
Filtered diastolic blood pressure (DAP) and muscle sympathetic nervous activity (MSNA) during a single low‐frequency baroreflex‐mediated cycle. The steepest decrease in DAP is shown as ×, and is a surrogate for the start of the blood pressure decrease stimulus. MSNA bursts are shown as red asterisks *, with three times the interquartile range shown as a dotted line to represent the prominence threshold. Stimulus time is the time from the steepest decrease in DAP to the first MSNA burst. Response time is the time from the last MSNA burst and the end of the cycle. DAP stimulus amplitude is the magnitude of DAP change from the beginning of the cycle to its lowest point. DAP response amplitude is the magnitude of DAP change from its lowest point to the end of the cycle. Here, the number of bursts is four, stimulus time = 1.50 sec, and response time = 1.99 sec. DAP stimulus amplitude = 10.5 mmHg, and response amplitude = 16.5 mmHg. The ratio of the 10.5 mmHg DAP stimulus to four MSNA bursts was used to compute the stimulus slope, and the ratio of four MSNA bursts to the 16.5 mmHg DAP response was used to compute the response slope.

For each of the 10 identified LF cycles, six indices were computed. These included four primary indices and two secondary indices (Table 1, Fig. 3). We first identified *D*_1_, *D*_2_, and *D*_3_, respectively, the first maximum, first minimum, and second maximum of each cycle. We then identified {*B*_*n*_}, the set of N MSNA bursts between *t*(*D*_1_) *and t*(*D*_3_), where *t* was time. Stimulus and response amplitudes were then defined, respectively, as *D*_1_ *–D*_2_ and *D*_3_ *–D*_2_. Stimulus and response times were defined, respectively, as *t*(*B*_1_)*–t*(*D*_1_) and *t*(*D*_3_)*–t*(*B*_*N*_). For each set of 10 stimulus amplitudes and times, the slope of the least squares linear regression was computed, meaning *f(a,b)* was minimized for $f\left(a,b\right)=\sum_{n=1}^{10}{\left(\left(ax_{n}+b\right)-y\left(x_{n}\right)\right)}^{2}$, where *x*_*n*_ were the amplitudes and *y* were the corresponding times. The response slope was computed analogously, with *x*_*n*_ the times and *y* the amplitudes.

**Table 1:** Names and definitions of the novel indices proposed

Primary indices (Fig. 3)	Stimulus time	Time from the steepest decrease in DAP to the first burst in a single LF cycle. This index is related to the time delay from decreases in DAP to subsequent sympathetic nerve activity at the vascular bed. The delay between carotid sinus nerve firing and muscle sympathetic nerve activity in the leg varies linearly according to the height of subjects and is ~2 sec (Hagbarth and Vallbo 1968; Borst and Karemaker 1983).
	Response time	Time from the last burst in a single LF cycle to the end of that cycle. This index is related to the time from the last norepinephrine release in the synaptic cleft to maximal arterial constriction. In a rat tail artery, norepinephrine clearance takes ≥1.5 sec (Gonon et al. 1993; Stjärne et al. 1994).
	Stimulus amplitude	The absolute difference between DAP at the beginning of a single LF cycle and its lowest point. This index is related to the magnitude of the acute blood pressure stimulus.
	Response amplitude	The absolute difference between DAP at the lowest point in a single LF baroreflex‐mediated cycle and its last point. This index is related to the magnitude of the neurogenic arterial constriction.
Secondary indices	Stimulus slope	The number of MSNA bursts within each LF cycle was plotted versus its respective DAP stimulus amplitude. A regression line was computed for each subject at each LBNP level using the method of least squares. For each subject, the stimulus slope is the slope of that line. This index quantifies the relationship between the amplitude of single acute blood pressure decrease to the subsequent MSNA. A large slope would mean that MSNA is not only triggered by acute decreases in DAP, but modulated by the size of that decrease.
	Response slope	DAP response amplitude was plotted versus the number of MSNA bursts within each respective LF cycle. A regression line was computed for each subject at each LBNP level using the method of least squares. For each subject, the response slope is the slope of that line. This index quantifies the relationship between MSNA and the subsequent rise in DAP. A large slope would mean that more intense MSNA causes larger LF increases in DAP. Indices similar to this response slope have been used, taking total change in MSNA and DAP into account, instead of individual LF cycles (Gulli et al. 2005b; Cooke et al. 2009).

The six proposed indices sought to apply the principles behind standard frequency domain baroreflex analysis, allowing their application to smaller, strictly defined time windows. For example, baroreflex gain essentially quantifies the amount to which variations in one variable occur simultaneously to variations in another. This was exactly what the stimulus and response slopes were designed to measure.

### Statistical analyses

Signal analysis was performed in Matlab 2014b (Mathworks, MA), and statistical analysis in JMP 11.2 (SAS Institute, Inc., NC). Values reported are mean ± 95% confidence interval. Confidence interval was computed as 1.95 the standard error within the group and LBNP level. Tukey's range test was used to compare values across LBNP levels and Student's *t* test was used to compare nonfinishers versus finishers at baseline. Values of *P* < 0.05 were considered significant.

## Results

Group data are presented for baseline, −10, −20, −30, and −40 mmHg LBNP, respectively. Group data are not shown for −50 and −60 mmHg because not all participants were able to tolerate these phases of testing due to imminent presyncope, with an associated reduction in statistical power.

Mean strength (*S*) for all cycles were 41.6 ± 6.2, 74.8 ± 10.5, 95.2 ± 13.9, 141.3 ± 24.7, and 211.3 ± 36.2 for increasing levels of LBNP. Mean strength for the 10 chosen cycles were 88.1 ± 11.9, 149.5 ± 24.3, 191.8 ± 31.7, 269.6 ± 40.8, and 426.7 ± 79.0. Units of *S* are *V*·*s*·*n*·mmHg.

Differences between the group of finishers and nonfinishers at baseline are detailed in Table 2. The average maximum coherence in the LF range between DAP and RR intervals was significantly lower in finishers (0.78 ± 0.22) compared to nonfinishers (0.92 ± 0.05), as was the LF gain between DAP and RR intervals (13 ± 3 vs. 21 ± 2 $\sqrt{\left(mmHg\cdot ms^{-1}\right)}$) and stimulus time (2.65 ± 0.66 vs. 3.88 ± 1.98 msec).

**Table 2:** Mean and standard deviation of cardiovascular indices for the two groups at baseline. The novel indices proposed are indicated in bold to distinguish them from the standard indices

	Baseline	
	Finishers	Nonfinishers
S (*V*·*s*·*n*·mmHg)	80.4 ± 42.7	97.3 ± 37.6
RR (msec)	938 ± 55	914 ± 191
SAP (mmHg)	115 ± 8	118 ± 17
DAP (mmHg)	55 ± 4	50 ± 3
SAPRR Coh (n.u.)	0.79 ± 0.17	0.92 ± 0.07
DAPRR Coh (n.u.)	0.78 ± 0.22	0.92 ± 0.05*
SAPRR gain, (mmHg/msec)^1/2^	12 ± 4	15 ± 3
DAPPRR gain, (mmHg/msec)^1/2^	13 ± 3	21 ± 2*
SAPRR freq (Hz)	0.093 ± 0.015	0.084 ± 0.022
DAPRR freq (Hz)	0.080 ± 0.010	0.083 ± 0.021
**Stim time (sec)**	2.65 ± 0.66	3.88 ± 1.98*
**Resp time(sec)**	3.21 ± 1.02	3.77 ± 0.56
**Stim amp (sec)**	8.12 ± 2.29	9.28 ± 1.53
**Resp amp (sec)**	8.72 ± 2.52	10.54 ± 2.98
**Stim slope (mmHg/n)**	0.02 ± 0.13	0.01 ± 0.15
**Resp slope (mmHg/n)**	0.98 ± 1.14	0.68 ± 1.28
**MSNA bursts/min**	10.49 ± 2.06	9.35 ± 5.69
**MSNA bursts/Hz**	60.68 ± 11.92	43.24 ± 18.78
**MSNA bursts/LF cycle**	2.75 ± 0.36	2.32 ± 0.93

Data from finishers (*n* = 6) and nonfinishers (*n* = 5). Values indicated with * are different from the value for the other group (*P* < 0.05).

**Figure 4:**
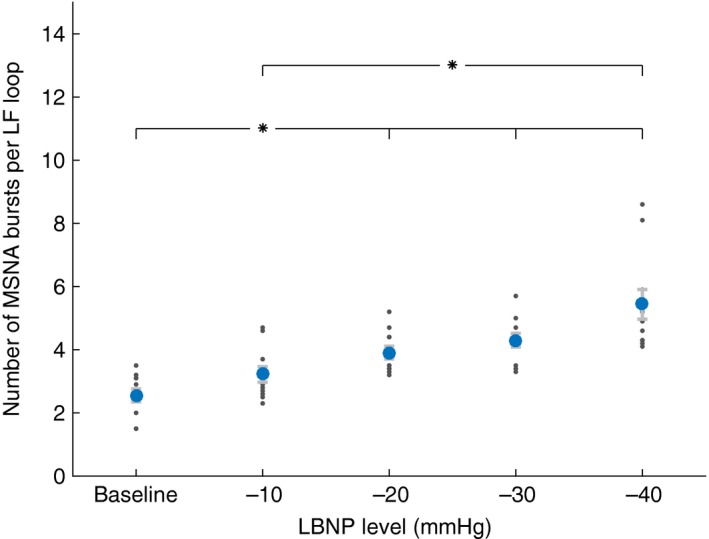
Muscle sympathetic nerve activity (MSNA) bursts within single low‐frequency baroreflex‐mediated cycles at baseline and during lower body negative pressure (LBNP) levels. For each level of LBNP, 10 low‐frequency baroreflex‐mediated cycles were chosen to maximize DAP amplitudes, area under MSNA, and number of MSNA bursts. Data are mean ± confidence interval (blue circles). For each participant, the mean of those 10 values are shown as smaller gray dots. *Indicates statistical difference between levels of LBNP with both groups taken together (*P* < 0.05).

**Figure 5:**
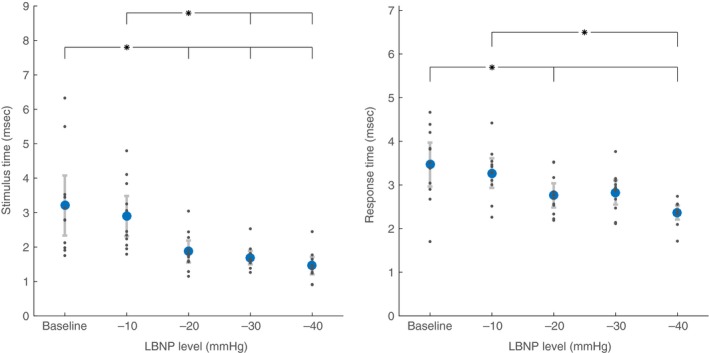
Stimulus and response times at baseline and during lower body negative pressure (LBNP). Within single low‐frequency baroreflex‐mediated cycles, stimulus time is the time from the steepest decrease in diastolic arterial pressure to the first muscle sympathetic nerve activity burst, and response time is the time from to last muscle sympathetic nerve activity burst to the end of the cycle. Data are mean ± confidence interval (blue circles). Data for each individual participant are shown as smaller gray dots. *Indicates statistical difference between levels of LBNP with both groups taken together (*P* < 0.05).

**Figure 6:**
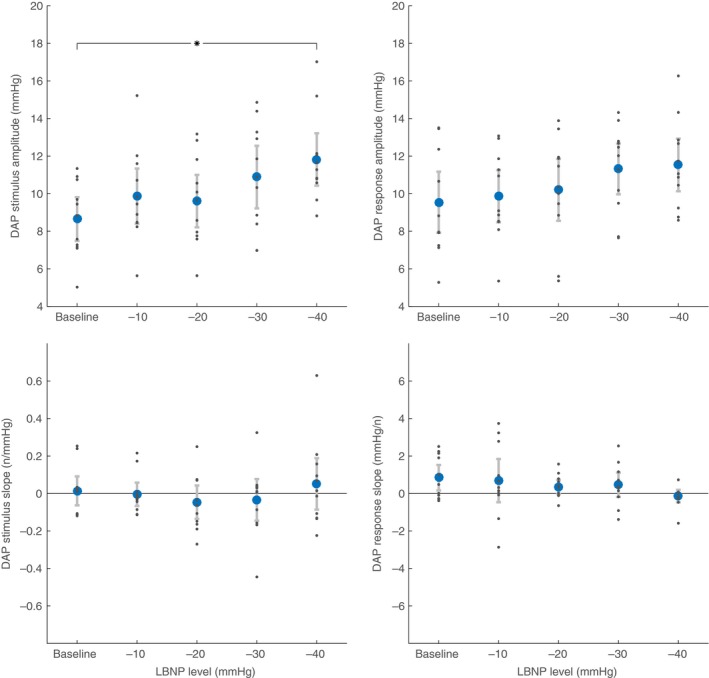
Diastolic arterial pressure (DAP) stimulus/response amplitudes and slopes at baseline and during lower body negative pressure (LBNP). Within single low‐frequency baroreflex‐mediated cycles, DAP stimulus and response amplitudes are the absolute difference between the lowest DAP in the cycle to the DAP at the beginning and end of the cycle, respectively. Stimulus and response slopes quantify the relationship between stimulus and response amplitude to the number of MSNA bursts within the same LF cycle. Data are mean ± confidence interval (blue circles). Data for each individual participant are shown as smaller gray dots. *Indicates statistical difference between levels of LBNP with both groups taken together (*P* < 0.05).

Differences between levels of LBNP, for all participants combined, are detailed in Table 3. Focusing on the indices developed in this study, increasing levels of LBNP significantly increased the number of MSNA bursts per LF cycle (Fig. 4), decreased both stimulus and response times (Fig. 5), and increased stimulus amplitude (Fig. 6).

**Table 3:** Mean and standard deviation of cardiovascular indices for all participants (*n* = 11). The novel indices proposed are indicated in bold to distinguish them from the standard indices

	Baseline	*−*10 mmHg	*−*20 mmHg	*−*30 mmHg	*−*40 mmHg
S (V·*s*·*n*·mmHg)	88 ± 39	149 ± 81	192 ± 105	270 ± 135*	427 ± 262*, †, ‡, §
RR (msec)	951 ± 132	938 ± 143	901 ± 144	832 ± 118	754 ± 114*, †
SAP (mmHg)	116 ± 12	113 ± 14	111 ± 12	108 ± 12	104 ± 11
DAP (mmHg)	53 ± 4	50 ± 5	50 ± 5	51 ± 6	51 ± 5
SAPRR Coh (n.u.)	0.85 ± 0.14	0.82 ± 0.14	0.86 ± 0.10	0.85 ± 0.09	0.87 ± 0.09
DAPRR Coh (n.u.)	0.84 ± 0.17	0.84 ± 0.07	0.87 ± 0.07	0.86 ± 0.08	0.87 ± 0.09
SAPRR gain, (mmHg/msec)^1/2^	14 ± 4	11 ± 3	10 ± 3	10 ± 5	8 ± 3*
DAPPRR gain, (mmHg/msec)^1/2^	16 ± 5	14 ± 4	13 ± 3	11 ± 3*	9 ± 3*, †
SAPRR freq (Hz)	0.089 ± 0.018	0.089 ± 0.024	0.094 ± 0.016	0.093 ± 0.019	0.083 ± 0.010
DAPRR freq (Hz)	0.081 ± 0.015	0.084 ± 0.018	0.093 ± 0.015	0.092 ± 0.018	0.088 ± 0.016
**Stim time (sec)**	3.21 ± 1.48	2.90 ± 0.99	1.87 ± 0.54*	1.69 ± 0.33*, †	1.46 ± 0.43*, †
**Resp time(sec)**	3.47 ± 0.86	3.27 ± 0.58	2.76 ± 0.47*	2.83 ± 0.47	2.37 ± 0.27*, †
**Stim amp (sec)**	8.7 ± 2.0	9.9 ± 2.5	9.6 ± 2.4	10.9 ± 2.8	11.8 ± 2.4*
**Resp amp (sec)**	9.6 ± 2.8	9.9 ± 2.4	10.2 ± 2.8	11.3 ± 2.3	11.5 ± 2.4
**Stim slope (mmHg/** ***n*** **)**	0.01 ± 0.13	−0.00 ± 0.11	−0.05 ± 0.15	−0.03 ± 0.19	0.05 ± 0.23
**Resp slope (mmHg/** ***n*** **)**	0.82 ± 1.15	0.69 ± 1.96	0.35 ± 0.64	0.47 ± 1.11	−0.14 ± 0.57
**MSNA bursts/min**	10.0 ± 3.9	12.9 ± 4.7	16.8 ± 3.5*	19.1 ± 2.8*, †	23.3 ± 6.3*, †, ‡
**MSNA bursts/Hz**	53 ± 17	58 ± 17	74 ± 10*	79 ± 14*, †	91 ± 33*, †, ‡
**MSNA bursts/LF cycle**	2.55 ± 0.68	3.22 ± 0.81	3.91 ± 0.67*	4.30 ± 0.72*	5.44 ± 1.56*, †

Data from finishers (*n* = 6) and nonfinishers (*n* = 5). Values indicated with *, †, ‡, and § are different from the value at baseline, −10, −20, and −30 mmHg, respectively (*P* < 0.05).

## Discussion

In this study, new signal analysis techniques were applied to isolate individual low‐frequency constriction–dilation cycles in DAP and MSNA; four primary indices and two secondary indices were defined. The primary goal was to investigate the relationship between DAP and MSNA during LBNP, with a secondary goal to identify differences in this relationship between the finishers and nonfinishers at baseline.

The mean number of MSNA bursts within single LF cycles increased as LBNP intensified. This expected behavior is consistent with escalating levels of cardiovascular stress increasing sympathetic outflow. Similar observations have been made previously, and its analog here suggests that our selection criteria and analysis of these chosen LF cycles capture important well‐known behaviors of the system. This increase in the number of MSNA bursts per LF cycle could also explain the corresponding increase in the strength index *S*, along with modest increases in stimulus/response amplitudes. However, we do not propose *S* as an index to quantify differences in baroreflex function between participants because it relies on the integral of the cycle's MSNA, which is affected by individual electrode placement. It is difficult to quantify the degree to which *S* was successful in ranking strongly coupled LF cycles within LBNP levels since no standard exists in this regard. The sensitivity of the subsequent indices to the way with which cycles were chosen with *S* could benefit from future detailed examination. Although variations in the number of cycles chosen caused only marginal changes in results, one could envision alternative approaches such as setting a threshold for *S* and keeping all cycles above this threshold (similarly to Marchi et al. 2016), or choosing a certain number of cycles containing one burst, two bursts, three bursts, etc., which might improve the quality of subsequent slope indices.

Stimulus time values were within the physiological range of delay between carotid sinus nerve firing and muscle sympathetic nerve activity in the leg (±2 sec; Hagbarth and Vallbo 1968; Borst and Karemaker 1983). They became progressively smaller with increasing LNBP, suggesting that as orthostatic stress increases, either the nervous system reacted more aggressively to progressively smaller decreases in DAP, and/or the reaction time between the DAP decrease stimulus and the MSNA response became shorter, that is, the system became either more sensitive or faster. In the first case, since the time delay was computed from a static hypothetical point on the DAP time series, a shift to the left of the actual point would cause a decrease in our estimate of the stimulus time index although the nervous processing and transmission time remained the same. In the second case, increased baroreceptor activity could reduce synaptic delays or open alternative pathways centrally, as well as recruit higher threshold/higher velocity neurons peripherally following a baroreflex analog to the well‐known motor‐unit recruitment “size principle” (Henneman and Mendell 2011). These two explanations are not mutually exclusive and our findings, which are consistent with other studies in this regard (Wallin et al. 1994; Macefield et al. 2002), do not favor one over the other.

Response time values were within the physiological range of norepinephrine clearance rate by neuronal uptake in the arterial bed (>1.5 sec (Stjärne et al. 1994; Gonon et al. 1993). As for stimulus time, response time became progressively smaller as LBNP intensified. The potential of this index to measure clearance by neuronal intake is of interest because this phenomenon is difficult to measure in vivo. This study's results suggest the existence of a stress adaptation mechanism capable of improving clearance rate. Interestingly, although previous research has shown norepinephrine transporter deficiency associated with orthostatic intolerance (Jacob et al. 1999; Shannon et al. 2000), we did not detect a significant difference in response time between groups at baseline. This may reflect that our nonfinishers did not necessarily have poor orthostatic tolerance, rather that the finishers were exceptional.

Nevertheless, baseline comparisons highlighted differences between groups for stimulus time, DAP‐RR gain, and DAP‐RR coherence. Although the values for SAP‐RR coherence were similar to those for DAP, the threshold for statistical significance was not quite reached. The gain and coherence results are consistent with other studies, the interpretation being that the baroreflex in individuals with lower orthostatic tolerance is active even in resting conditions, perhaps compensating for deficiency in other mechanisms. A longer stimulus time in the nonfinishers group might also indicate a relative baroreflex impairment (longer response times in the face of decreases in pressure would impair blood pressure regulation). Indeed, there are reports that increased baroreflex delay is associated with poorer orthostatic tolerance (Gulli et al. 2003, 2005a,b). Interestingly, we also saw a trend for a lower central frequency of the SAP‐RR cross‐spectral analyses in the nonfinishers, which is comparable with previous reports (Gulli et al. 2003) and also associated with a greater baroreflex time delay in those with poorer orthostatic tolerance.

No differences were found in the slope indices either between groups at baseline or between LBNP levels. These indices quantify the degree to which large acute decreases in DAP cause high MSNA, which in turn cause large acute rises in DAP. Our results suggest that these variables are independent, whereby although sequences of MSNA bursts are caused by acute decreases in DAP, their numbers and amplitude might be modulated by a longer term blood pressure moving average.

The idea of identifying individual sequences of increasing/decreasing DAP and associating them with analogous sequences in MSNA burst rate was recently used to quantify sympathetic baroreflex sensitivity (Marchi et al. 2016). Although our methods distinguish themselves through the identification of individual LF cycles, as well as the separation of decreasing versus increasing sequences of DAP, the publication reasserts the need to quantify the sympathetic baroreflex without the limitations incurred by frequency domain computations.

The six indices proposed by this study could perhaps be used to enhance the knowledge already attained by cross‐spectral methods by better elucidating the point within the baroreflex arc that is most affected by pathology or interventions.

### Limitations

The clearest limitation of the study was its low number of participants (finishers *n* = 6, nonfinishers *n* = 5), which has an obvious effect on statistical power. Furthermore, although reasonable variations of the parameters used for the automatic identification of the signal features produced equivalent results, the use of these techniques to obtain clear physiological conclusions would necessitate further validation and simulations.

It should be noted that MSNA is a technique that is all but absent in clinical settings, and demands often prohibitively high levels of expertise in laboratory settings.

The fact that only male participants were included importantly limits the scope of the study, especially given that known differences exist between male and female baroreflex mechanisms (Laitinen et al. 1998). It would also be of interest to perform these measurements on patients suffering from orthostatic intolerance or those with hypertension.

### Conclusion

The proposed methodologies used intuitive techniques to compare direct amplitudes and time delays within individual neurogenic LF baroreflex cycles. They offer an interesting complement to accepted cross‐spectral analysis methods, and help clarify the role of the different parts of the closed baroreflex loop. Further exploration of sympathogenic fluctuations with these indices could provide information to derive new techniques to quantify processes in the arterial branch of the baroreflex, which could be used to diagnose and monitor patients with disorders of baroreflex function.

## Conflict of Interest

None declared.
